# Effect of vatinoxan co-administered with medetomidine on intraocular pressure and pupil diameter in healthy dogs

**DOI:** 10.17221/47/2025-VETMED

**Published:** 2025-12-19

**Authors:** Petr Rauser, Marketa Mrazova, Alena Sabatova, Karolina Jiraskova

**Affiliations:** Department of Surgery and Orthopedics, Small Animal Clinic, Faculty of Veterinary Medicine, University of Veterinary Sciences, Brno, Czech Republic

**Keywords:** alpha-2 adrenoreceptor agonist, alpha-2 adrenoreceptor antagonist, MK-467, L-659,066, pupil size

## Abstract

Medetomidine, an alpha-2 adrenoreceptor agonist, is used for sedation. This study aimed to determine the influence of the alpha-2 adrenoreceptor antagonist vatinoxan, co-administered with medetomidine, on healthy dogs’ intraocular pressure (IOP) and pupil diameter (PD). A prospective, randomised, masked clinical study was performed. A total of 40 conscious dogs were allocated to one of two groups: medetomidine 0.01 mg/kg with vatinoxan 0.2 mg/kg intravenously (MV-group, *n = *20) or medetomidine 0.01 mg/kg intravenously (M-group, *n* = 20). The IOP, PD, heart rate, and mean arterial pressure were measured prior to baseline and 5, 10, and 20 min after drug administration. Data were analysed using one- and two-way repeated measures ANOVA or their non-parametric equivalents (*P* < 0.05). No significant differences in IOP within or between groups were recorded. In the MV-group, PD remained unchanged compared to baseline. In the M-group, PD significantly (*P* = 0.007, *P* < 0.001, *P* < 0.001) decreased compared to baseline at all observation times. PD was significantly (*P* = 0.010, *P* < 0.001, *P* < 0.001) smaller in the M-group at all observation times compared to the MV-group. Vatinoxan co-administered with medetomidine, as well as medetomidine alone, did not significantly influence IOP. Vatinoxan with medetomidine did not affect PD, while medetomidine alone significantly reduced it.

Intraocular pressure (IOP) plays an important role in ocular homeostasis. Its changes can have a significant influence on eye function. The IOP is generated by the pressure of the aqueous humour on the cornea and sclera and is influenced by changes in the volume of the aqueous humour, choroid, and vitreous, scleral rigidity, extraocular muscle tone, and external pressure on the globe (Almeida et al. 2004).

The IOP is also affected by pressure on the eye globe, arterial carbon dioxide and oxygen tensions, systemic blood pressure, or drugs administered (Wrezesniewska et al. 2018). The IOP is also influenced by the pupil diameter (PD) – miosis reduces IOP by increasing the aqueous humour outflow (Johnstone et al. 2021). Normal intraocular pressure in the dog is approximately 10–25 mm Hg (Renwick 2002).

Medetomidine is an alpha-2 adrenoceptor agonist commonly used in small animal anaesthesia for sedation or anaesthesia premedication. It induces sedation through central alpha-2 adrenoceptor agonism and has significant peripherally and centrally mediated hemodynamic side effects. The initial increase in blood pressure results from peripheral vasoconstriction caused by activation of post-synaptic alpha-2 receptors in peripheral vascular smooth muscles. This is associated with increased vagal tone and decreased heart rate (Murrell and Hellebrekers 2005).

The alpha-2 adrenoceptor antagonist vatinoxan (MK-467, L-659,066) only poorly penetrates the blood-brain barrier in dogs and was shown to be peripherally selective after co-administration with medetomidine (Honkavaara et al. 2020). Vatinoxan reduces peripherally mediated vasoconstriction and bradycardia while maintaining central sedative action (Rolfe et al. 2012).

Medetomidine has a minimal effect on IOP (Kanda et al. 2015). However, the influence of medetomidine in combination with vatinoxan on IOP has only been mentioned in the Thesis of Bertoni (2024), but the effects on PD have not been described. The purpose of this study was to investigate the 20-minute influence of vatinoxan co-administered intravenously with medetomidine on IOP and PD in healthy conscious dogs. We hypothesise that the effects of medetomidine with vatinoxan will be similar to those of medetomidine alone, without significantly affecting IOP or PD.

## MATERIAL AND METHODS

### Study design

This prospective randomised masked clinical study was performed with the consent of the Ethical Committee of the University of Veterinary Sciences Brno. All owners provided informed consent to have their animals participate in the study.

### Study animals

A total of 40 healthy dogs aged 2–8 years and weighing 5–20 kg undergoing musculoskeletal radiography under sedation were included. All dogs were classified as healthy based on medical history, physical examination, and complete blood counts and biochemical profiles. The dogs were fasted for 6 h, with free access to water. An experienced ophthalmologist performed an ophthalmic examination. Only dogs without eye abnormalities were included, with an IOP measured at 15–25 mm Hg and a Schirmer Tear Test I score higher than 12 mm/minute. This study did not include dogs of brachycephalic breeds, Cocker Spaniels, Miniature Schnauzers, or Terriers. All the measurements were performed in conscious animals.

### Procedure

A randomising software (www.randomizer.org) was used to allocate the animals into the MV-group (*n* = 20) or the M-group (*n = *20). An intravenous (i.v.) cannula was placed in the cephalic vein in all dogs. After 10 min, the intraocular pressure (IOP), pupil diameter (PD), heart rate (HR), and mean arterial pressure (MAP) were measured and recorded (baseline). For sedation, dogs of the MV-group received medetomidine at a dose of 0.01 mg/kg, with vatinoxan at a dose of 0.2 mg/kg i.v. (Zenalpha, Vetcare, Finland), and dogs of the M-group received medetomidine at a dose of 0.01 mg/kg i.v. (Domitor, Orion, Finland). Drugs were injected through the cannula within 5 seconds. The cannula was flushed with 3 ml of saline.

All data were collected in the morning after a 20-minute acclimatisation period in a quiet room without windows, under constant light conditions. All the dogs were maintained in sternal recumbency throughout the study period without compression on the jugular vein or eye globe. The same person performed the measurements, unaware of which drug had been administered.

In all the dogs, IOP, PD, HR, and MAP were continuously measured and recorded immediately before (baseline) and 5, 10, and 20 min after i.v. administration of the drugs. The IOP was measured using applanation tonometry (TonoPen XL, Medtronic, MN, USA). Before the measurement, a new rubber cover was placed, and the tonometer was calibrated. The IOP was measured only on the left eye in all the animals. Three measurements with a 5% error and a maximum variance of 10% were averaged. The PD was measured only on the left eye in all the dogs using a pupilometer (Haab’s pupilometer; Merck Sharp & Dohme, NJ, USA). The HR was measured by auscultation of heart sounds over 20 s and multiplied by three. The MAP was measured non-invasively using a vital function monitor (Cardel 9401; Midmark, OH, USA) by a cuff applied to the antebrachium of the left forelimb. Cuff width was 40% of the circumference of the limb.

### Statistical analysis

A sample size calculation was performed after recruiting the first 20 dogs, 10 in each group. Because IOP remained unchanged, PD was used to determine the minimum sample size. It was determined that 16 dogs in each group would be required for the study to have an 80% power to detect a difference (α = 0.05) in mean PD of 2 mm between MV and M-groups at 20 min following drug administration. The clincalc.com software was used to calculate the sample size.

Normality was tested by the Shapiro–Wilk test. Normally distributed data were reported as mean ± standard deviation, data with non-normal distribution as median (minimum–maximum). The data with normal distribution measured at 5, 10, and 20 min were compared to the baseline using a one-way ANOVA for repeated measures with Bonferroni correction. The Friedman test was used to assess the non-normally distributed data. The variables were compared between groups at each specific time point using 2-way ANOVA for normally distributed data or the Wilcoxon test for non-normally distributed data (*P* < 0.05).

## RESULTS

A total of 13 breeds, 22 males and 18 females, aged 5.0 ± 2.4 years, and weighing 11.9 ± 5.8 kg, were included. In the MV-group, IOP decreased in 7 dogs, increased in 4 dogs, first increasing and then decreasing in 1 dog, first decreasing and then increasing in 1 dog, and remained unchanged in 7 dogs. In the M-group, IOP decreased in 5 dogs, increased in 4 dogs, first increased and then decreased in 3 dogs, first decreased and then increased in 1 dog, and remained unchanged in 7 dogs. No significant differences were observed in IOP values within or between the groups at any time points (Table 1).

**Table 1:** Changes in intraocular pressure (IOP), pupil diameter (PD), heart rate (HR) and mean arterial pressures (MAP) in healthy dogs before (baseline) and 5, 10 and 20 min after intravenous administration of medetomidine at a dose 0.01 mg/kg with vatinoxan at a dose 0.2 mg/kg (MV group) or medetomidine at a dose 0.01 mg/kg (M group)

Study groups	Variable	Time (min)			
		baseline	5	10	20
MV-group	IOP (mm Hg)	21 ± 3	21 ± 4	21 ± 4	20 ± 4
	PD (mm)	6 (4–7)	6 (4–8)	6 (4–7)	6 (4–7)
	HR (beats per minute)	117 ± 14	88 ± 20 (*P <* 0.001)*	82 ± 18 (*P <* 0.001)*	69 ± 17 (*P <* 0.001)*
	MAP (mm Hg)	132 ± 25	114 ± 24	113 ± 23 (*P** = ***0.044)*	99 ± 24 (*P** = ***0.003)*
M-group	IOP (mm Hg)	19 ± 3	20 ± 3	19 ± 3	19 ± 8
	PD (mm)	6 (5–7)	4 (3–6) (*P** = ***0.007)*; (*P*** = **0.010)^†^	4 (2–6) (*P <* 0.001)*; (*P <* 0.001)^†^	2 (1–5) (*P <* 0.001)*; (*P <* 0.001)^†^
	HR (beats per minute)	110 ± 23	67 ± 30 (*P** = ***0.001)*; (*P*** = **0.018)^†^	64 ± 21 (*P <* 0.001)*; (*P*** = **0.009)^†^	51 ± 25 (*P <* 0.001)*; (*P*** = **0.037)^†^
	MAP (mm Hg)	129 ± 35	118 ± 35	120 ± 33	115 ± 19 (*P** = ***0.007)*; (*P*** = **0.033)^†^

*Significantly lower compared to baseline; ^†^Significantly smaller (PD) or lower (HR, MAP) compared to MV group

Data are expressed as mean ± standard deviation for data with normal distribution or median (range) for data with not-normal distribution

In the MV-group, PD decreased in 5 dogs, increased in 7 dogs, and remained unchanged in 7 dogs. In 1 dog of MV-group, the PD first decreased and then increased. In the MV-group, PD did not change significantly compared to baseline. In the M-group, PD decreased in all dogs. In the M-group, PD significantly decreased at 5, 10, and 20 min compared to baseline. In the M-group, PD was significantly smaller compared to the MV-group at 5, 10, and 20 min (Table 1).

The HR significantly decreased in both groups at 5, 10, and 20 min compared to baseline. In the MV-group, HR was significantly higher compared to the M-group at 5, 10, and 20 minutes. In the MV-group, MAP significantly decreased at 10 and 20 min compared to baseline. In the M-group, MAP significantly decreased at 20 min compared to baseline. In the MV-group, MAP was significantly lower compared to the M-group at 20 min (Table 1). No other significant changes within or between groups were detected.

## DISCUSSION

Medetomidine decreases IOP by reduction in aqueous humour production caused by the activation of pre-junctional alpha-2 adrenoceptors and/or by inhibition of adenylyl-cyclase by the activation of epithelial alpha-2 adrenoceptors, or by vasoconstriction in the ciliary body triggered by activation of post-junctional alpha-2 adrenoceptors (Kanda et al. 2015). However, in our dogs, we did not detect a decrease in IOP after the administration of medetomidine, which corresponds with previously published studies (Verbruggen et al. 2000; Wallin-Hakanson and Wallin-Hakanson 2001; Kanda et al. 2015; Mrazova et al. 2018). The reason may be the low dose of medetomidine we used. Bertoni (2024), in his Thesis, compared the effects of medetomidine alone and the combination of medetomidine with vatinoxan, both administered in identical doses as in our study on IOP. He noted a decrease in IOP in both groups. Combining vatinoxan and medetomidine resulted in a less pronounced reduction in IOP than medetomidine alone (Bertoni 2024). However, we did not detect a decrease in IOP in our dogs even after administering medetomidine with vatinoxan.

Pupillary constriction increases aqueous humour outflow through the trabecular meshwork, reducing IOP (Gelatt and Brooks 1999). The suppression of sympathetic activity induced by alpha-2 adrenoceptor agonists inhibits constriction of the iris dilator muscle, which is innervated primarily by the sympathetic nervous system (SNS). However, this inhibition does not induce miosis; it only inhibits mydriasis. The iris sphincter muscle is innervated not only by the parasympathetic nervous system (PNS), but also by the SNS (Randall 1999). Suppression of adrenergic nerve activity may also suppress the inhibition of the PNS cholinergic nerves that innervate the iris sphincter muscle. To date, no report has completely elucidated the mechanism of miosis induced by alpha-2 adrenoceptor agonists in dogs (Kanda et al. 2015).

There are conflicting reports about the effect of medetomidine on the PD – both mydriasis and miosis have been reported. Verbruggen et al. (2000) or Mrazova et al. (2018) observed a significant decrease in PD after intravenous injection of medetomidine, similar to what we did in our study. However, neither Kanda et al. (2015) nor Aghababaei et al. (2021) detected a significant change in PD. After administration of medetomidine with vatinoxan, we did not observe a marked reduction of PD. This may be due to the effects of vatinoxan, which partially limited the impact of the alpha-2 adrenoreceptor agonist on the pupil.

Miosis reduces IOP by increasing the aqueous humour outflow (Johnstone et al. 2021). We would expect dogs with significantly lower PD after administration of medetomidine to have lower IOP compared to dogs after injection of medetomidine with vatinoxan. However, IOP was not significantly affected in either group. We therefore assume that the reduction in aqueous humour production in the ciliary body after the administration of alpha-2 adrenoreceptor agonist is more important for influencing IOP than the increase in aqueous humour outflow induced by miosis.

The effect of medetomidine on PD may make it difficult to obtain sufficient mydriasis for ophthalmoscopy, electroretinography, and/or intraocular surgery. For these purposes, medetomidine with vatinoxan should be more suitable, since it does not induce such a significant reduction of PD.

This study has several limitations: the small number of dogs made excluding a statistical type II error impossible. However, several studies using small numbers of animals have shown significant clinical findings. Our study used only one dose of medetomidine alone or with vatinoxan. If higher doses of medetomidine were used, as in the study by Kanda et al. (2015), or if a different ratio of medetomidine to vatinoxan had been used, the results might have been different. Another limitation of our study is the observation period. We were time-limited because medetomidine or medetomidine with vatinoxan were administered in clinical patients. A more extended observation period would be necessary for more valuable conclusions. The present study was performed in healthy dogs. However, further studies should be conducted on the effect of medetomidine with vatinoxan on IOP and PD in dogs with pre-existing systemic and/or ocular disease.

In healthy dogs, medetomidine alone or with vatinoxan in the ratio and dose used did not significantly influence intraocular pressure. Medetomidine with vatinoxan did not affect pupil diameter, while medetomidine alone considerably reduced it.
